# Supplementary material for: Order-disorder phase transition driven by interlayer sliding in lead iodides
Source: Nat Commun. 2023 Apr 8;14:1981. doi: 10.1038/s41467-023-37740-1 (PMC10082779; doi:10.1038/s41467-023-37740-1)
Supplement: Supplementary file 1 — Supplementary information [file 41467_2023_37740_MOESM1_ESM.pdf]

Supplementary information

Order-disorder phase transition driven by interlayer sliding in lead iodides

Seyeong Cha *et al.*

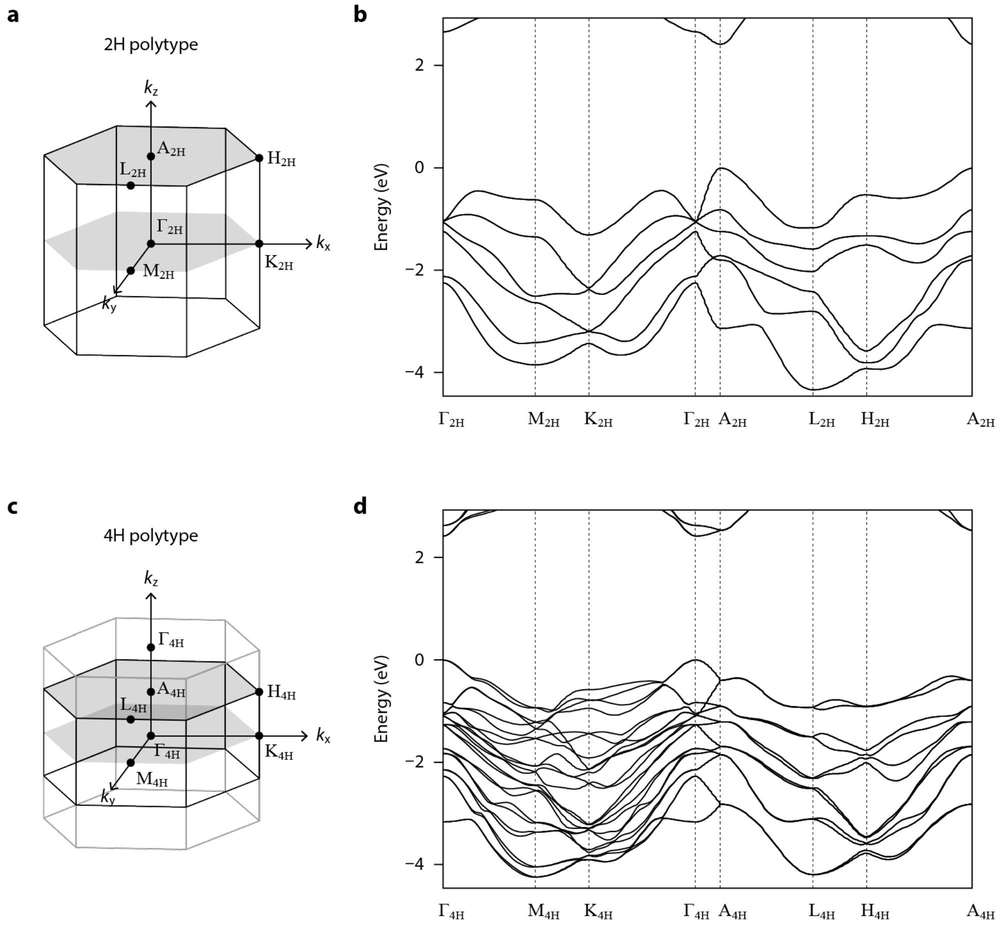

Fig. 1 | Comparison of bulk Brillouin zones and band structures between 2H-PbI_2 and 4H-PbI_2. a Bulk Brillouin zone of 2H-PbI_2 in the conventional nomenclature of PbI_2 (1T in the nomenclature of TMDs). The closed circles mark high symmetry points, and grey regions are high-symmetry planes. **b** Band structure of 2H-PbI_2 calculated by DFT, considering spin-orbit coupling. The VBM is located not at the Γ_{2H} point, but at the A_{2H} point. **c** Bulk Brillouin zone of 4H-PbI_2 (2H in the nomenclature of TMDs), compared to that of 2H-PbI_2 (grey lines). The A_{2H} point in **a** is overlapped with the second Γ_{4H} point. **d** Band structure of 4H-PbI_2 calculated by DFT, considering spin-orbit coupling. The VBM of 4H-PbI_2 is located at the Γ_{4H} point.

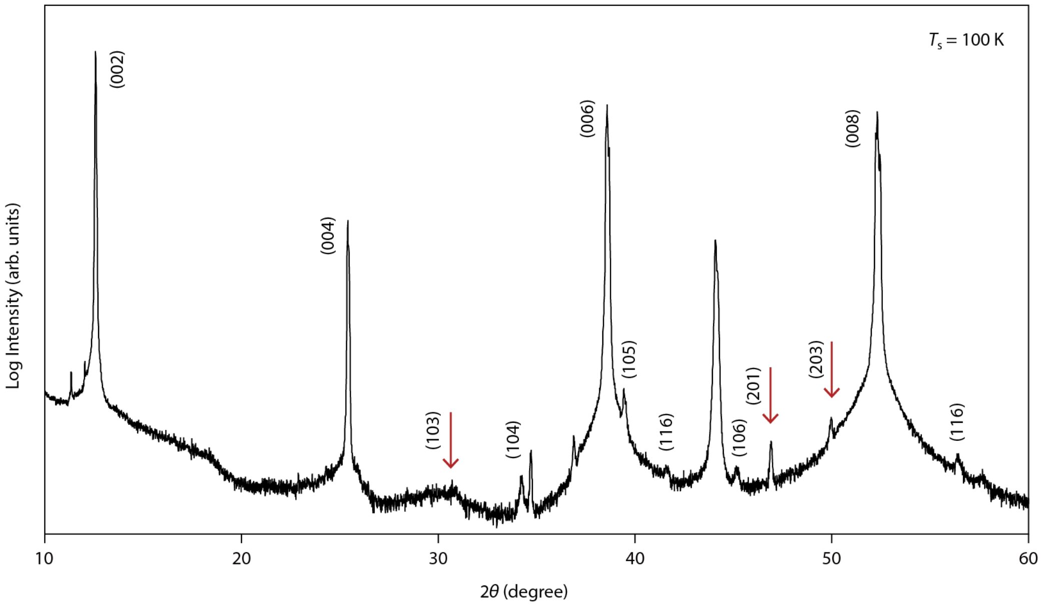

Fig. 2 | XRD measurements. XRD data taken from our single-crystal PbI_2 samples at 100 K and plotted as a function of 2θ. Red arrows indicate the peaks expected for the 4H phase (ICDD card no. 04-007-3144), which is consistent with the previous XRD reports[32,33].

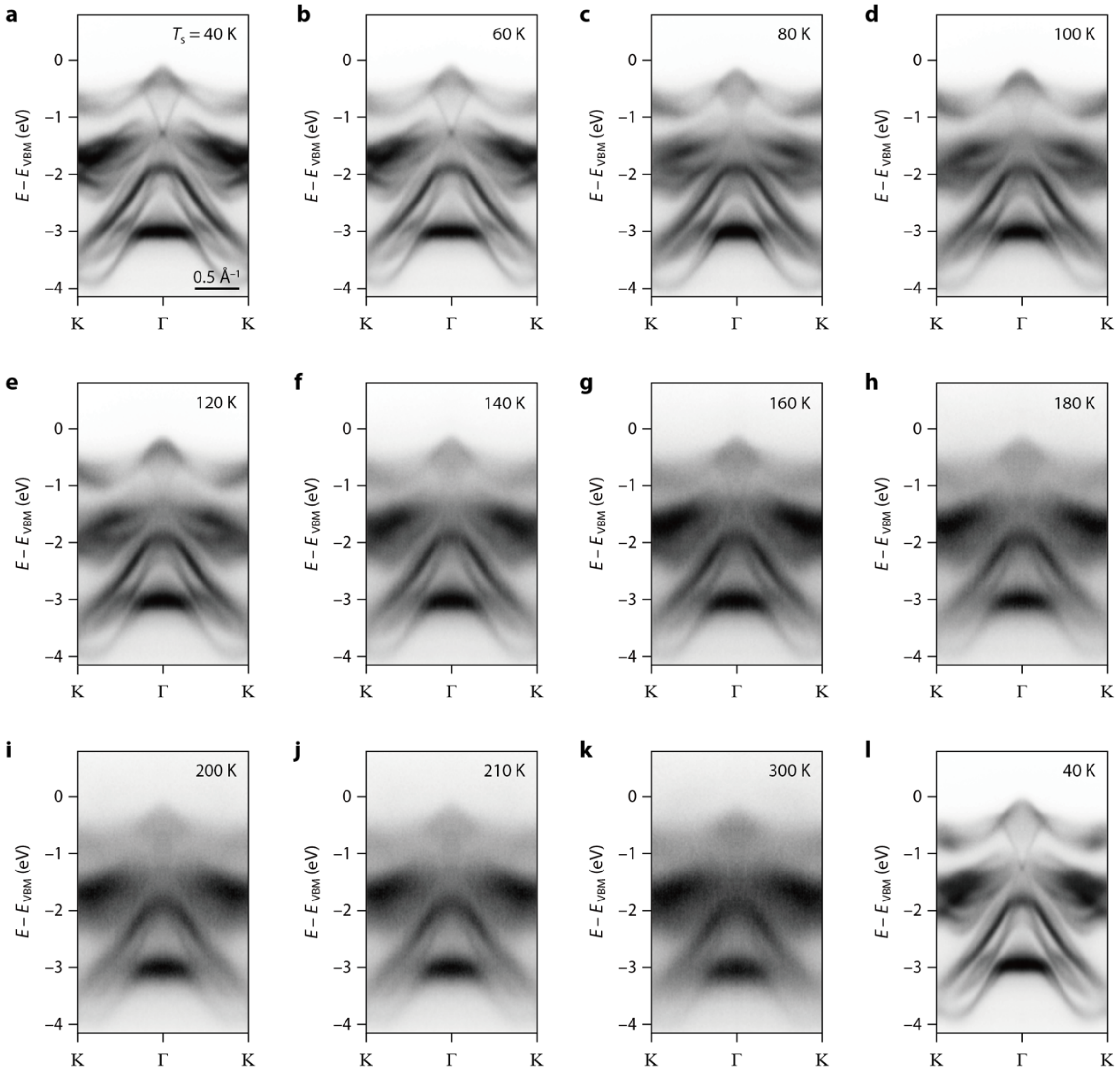

Fig. 3 | Complete set of data and the reversibility of phase transitions. a-l ARPES data taken at the same experimental conditions but T_S marked on the upper right of each panel. Those in **a-k** were taken as increasing T_S, and that in **l** was taken after cooling the sample down to 40 K. One can clearly see that the spectral broadening of valence bands induced by the phase transition above T_C returns to its original state as cooling samples down below T_C, confirming that this phase transition is reversible.

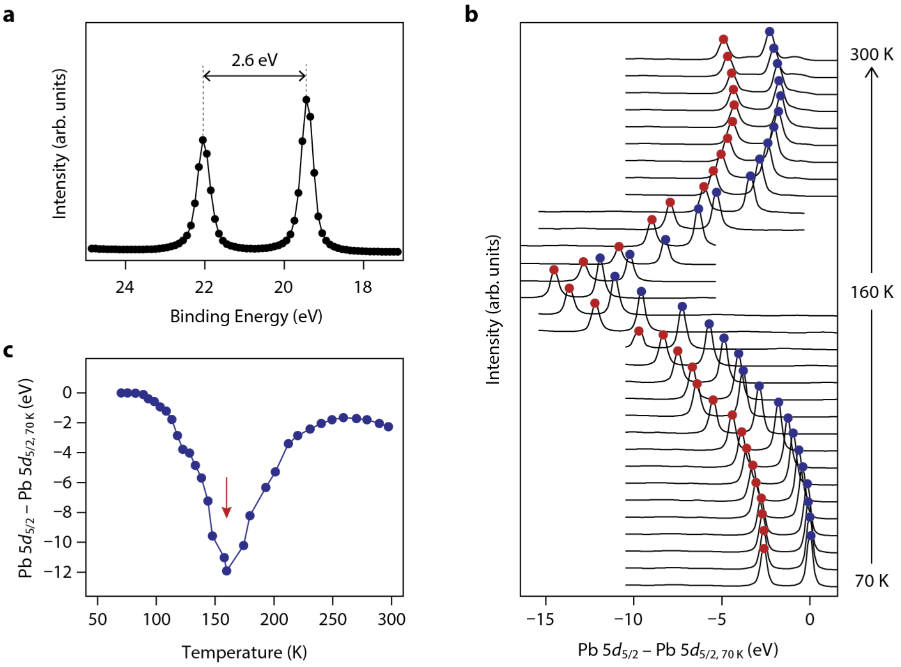

Fig. 4 | Core-level photoemission and temperature dependence of charging effects. a Pb 5*d* core-level spectra of bulk PbI_2 taken with the photon energy of 96 eV at T_S = 70 K. The spin-orbit splitting of the Pb 5*d* doublet is estimated to be about 2.6 eV. **b** Temperature dependence of Pb 5*d* core-level spectra from 70 K to 300 K as marked on the right. Little is changed in the line shape except for thermal broadening, but the kinetic energy of Pb 5*d* photoelectrons shows strong temperature-dependent changes due to the charging effects. **c** Relative kinetic energy of Pb $5d_{5/2}$ peaks with respect to that at 70 K is plotted as a function of T_S. It shows the dip in the vicinity of 160 K as indicated by the red arrow, which is consistent with photocurrent versus T_S in Fig. 2f. This is another manifestation of the highly insulting state above T_C probably because of phase fluctuations.

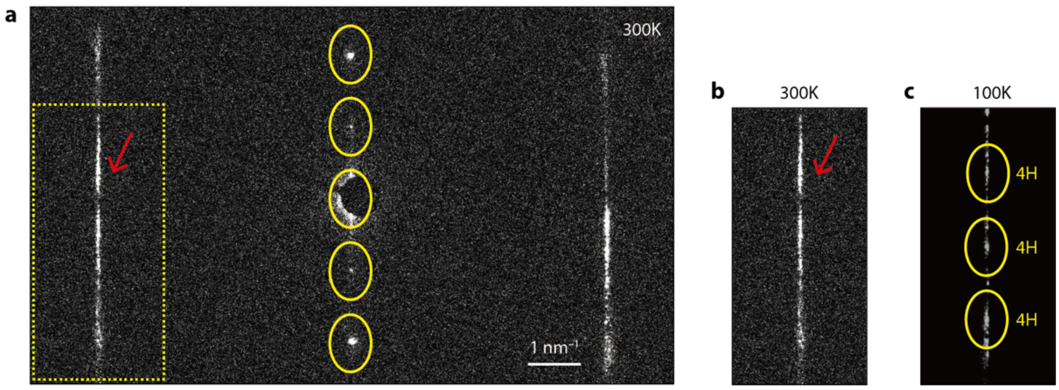

Fig. 5 | Diffraction pattern in the ⟨110⟩ direction. a SAED patterns of PbI_2 taken for the larger area of 80 × 80 nm^2 at 300 K along the *y* axis. **b, c** Part of SAED patterns indicated by the yellow dotted box in **a**, taken at (**b**) 300 K and (**c**) 100 K. The yellow ovals mark the spotty 4H-PbI_2 peaks, and the red arrows indicate the formation of streaks, which is clear evidence for disorder or the randomness of interlayer sliding.

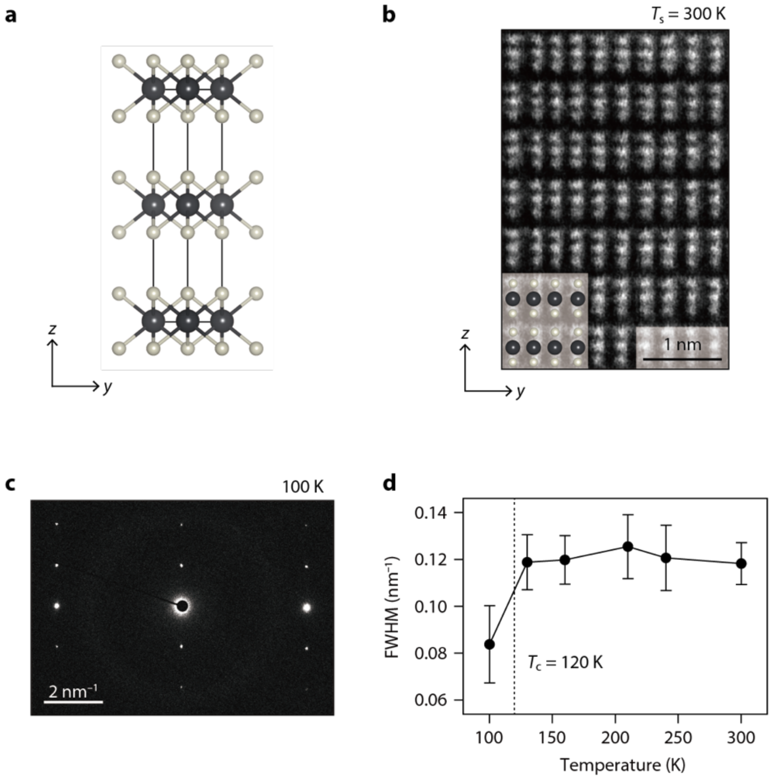

Fig. 6 | Diffraction patterns in the ⟨100⟩ direction. a Ball-and-stick model of 4H polytype PbI_2 viewed from the *x* axis over the *yz* plane. The black balls represent Pb atoms, and white balls represent I atoms. The black rectangle overlaid is the unit cell. **b** HAADF-STEM images of PbI_2 at $T_s = 300$ K taken along the *x* axis. For this relatively small area of 3×4.5 nm^2, one of the 3 shifts can be dominant owing to the nanoscale triple-domain separations arising from remnant interlayer coupling at the intermediate phases between the ordered and the fully disordered phases. No sign of interlayer sliding is found in the *y* direction or, more generally, ⟨100⟩, which not only rules out the possibility of electron-beam effects, but also indicates strong in-plane anisotropy. **c** SAED pattern of PbI_2 taken along the *x* axis. **d** FWHM of SAED peaks taken along the *z* direction as a function of temperature. This result demonstrates that the signature of interlayer sliding above T_c can also be captured in SAED patterns taken over the wide area of 80×80 nm^2 towards the formation of steaks as observed in Fig. 3e, f.
